# Trends in Antimicrobial Usage on Swiss Pig Farms from 2018 to 2021: Based on an Electronic Treatment Journal

**DOI:** 10.3390/antibiotics13090831

**Published:** 2024-09-02

**Authors:** Ramona Wissmann, Dolf Kümmerlen, Thomas Echtermann

**Affiliations:** Division of Swine Medicine, Department for Farm Animals, Vetsuisse Faculty, University of Zurich, 8057 Zurich, Switzerland

**Keywords:** pig, swine, antimicrobial usage, treatment incidence, defined daily doses, used daily doses, Highest Priority Critically Important Antimicrobials

## Abstract

(1) Background: The aim of this retrospective observational study was to observe the trends in antimicrobial usage (AMU) from 2018 to 2021 in Swiss pigs based on an electronic treatment journal used nationwide by farmers. Thus, for the first time, standardized, longitudinal comparisons of AMU between the years could be analyzed, as well as the influence of targeted interventions, on farms with higher consumption. (2) Methods: The data was evaluated by different indicators, such as the amount of active ingredient in kilograms, treatment days per farm (ATI) and treatment incidence (TI) based either on animal-defined daily doses (TIADD) or used daily doses (TIUDD). Calculations were performed across the following five age categories: suckling piglets, weaners, fattening pigs, and gestating and lactating sows, and the proportions of antimicrobial classes were evaluated for each age category. (3) Results: The highest amount of the active ingredient was administered to the group of fattening pigs, while the suckling piglets received the lowest amount of the active ingredient. In 2021, there was a significant decrease in active ingredient consumption per pig, but a significant increase in ATI, TIADD and TIUDD compared to 2018. The largest proportion of AMU was attributed to penicillins each year, followed by sulfonamides and tetracyclines. The “Highest Priority Critically Important Antimicrobials” represented a proportion of overall usage, declining from 5.2% in 2018 to 3.1% in 2021, while polypeptides were the most used class of critical antimicrobials. Interventions on high-usage farms showed that some farms decreased their AMU in the following year while others did not. (4) Conclusions: This study reveals a decrease in the overall usage measured in kilograms per pig of antimicrobials in Swiss pigs between 2019 and 2021 through the monitoring of AMU, but, at the same time, there was an increase in treatment days or incidence per farm. Critical antimicrobials can be reduced regardless of the indicator. The significance and quality of interventions should be investigated in future studies.

## 1. Introduction

Antimicrobials are a vital tool for the treatment and prevention of bacterial infections in both human and veterinary medicine. Since bacteria exist, they adapt and develop resistance to survive in unfavorable conditions [1]. One of the primary causes for the emergence and spread of antimicrobial resistance (AMR) is the (mis)use of antimicrobial substances in agriculture and veterinary and human medicine [2,3,4,5].

The Highest Priority Critically Important Antimicrobials (HPCIAs), such as third-, fourth- and fifth-generation cephalosporins, fluoroquinolones and macrolides, and, since 2017, polypeptides (in pigs mainly represented as colistin), are of particular importance for the treatment of diseases in human medicine [6]. The link between the use of fluoroquinolones, for example, and the prevalence of resistance to this active substance in Swiss pigs and in the pig environment is well documented [7,8]. Therefore, HPCIAs are no longer allowed to be dispensed in stock on Swiss farms due to a revision of the Ordinance on Veterinary Medicinal Products in 2016, and their usage requires documented and defined indications such as the treatment failure of a non-HPCIA antimicrobial [9].

The reduction in antimicrobial usage (AMU) is based on meaningful monitoring programs. Various European countries have introduced monitoring programs to track AMU (for further detail, refer to https://aacting.org/monitoring-systems/, accessed on 1 July 2024), such as the “yellow card” system in Denmark. In Switzerland, the monitoring system is called the “SuisSano/Safety + Health program”, which is provided by the Swiss Pig Health Services and requires the registration of all treatment data in an electronic treatment journal (ETJ). Among other nationwide programs, the ETJ’s AMU monitoring documents the antimicrobials that farmers administer to their animals, ensuring the correct dosage and duration of treatment for the animals [10]. This contrasts with other programs that work with either pre-defined doses and treatment durations or those prescribed by a veterinarian. In addition, within the ETJ, the well-established and scientifically proven indicators’ quantities of active ingredient in kilograms, treatment days per farm (ATI) and treatment incidence (TIADD and TIUDD) can be analyzed side-by-side at the same time [11]. Since 2020, participation in the program has been mandatory for all Swiss pig farms, and so far, there is no data on how the AMU monitored within this program has developed over the last few years.

Based on the monitoring, herd-specific intervention is possible and has shown a decreasing effect on AMU in several countries [12], including Denmark, where the introduction of the yellow card system has led to a reduction in AMU [13,14]. High-usage farms are identified and supported by a phone call or a farm visit. A study conducted one year after the introduction of the Swiss monitoring system determined the impact of the program, and no overall reduction in AMU could be shown [10], but detailed data on the effect of the program from its beginning till today are missing.

Therefore, the aim of this retrospective observational study was (i) to observe the (longitudinal) development of the AMU from 2018 to 2021 in Swiss pigs based on the measurements of the ETJ and (ii) to analyze the effect of the specific herd interventions within this program. To evaluate the AMU, the amounts of active ingredient in kilograms, treatment days per farm and treatment incidence (TIADD and TIUDD) were used as indicators.

## 2. Results

Over the years, the number of farms enrolled in the SuisSano/Safety + Health program has consistently increased (Table 1). In 2018, 1’213 farms participated, with 1160 of them using antimicrobials. Subsequently, in 2019, of 1526 farms, 1480 used antimicrobials. In 2020, the program registered 2339 participating farms, 2225 of which were antimicrobial users. Finally, in 2021, the program had 3539 farms enlisted, of which 3288 used antimicrobials.

Like the number of farms, the number of pigs per age category increased yearly within the study period from 2018 to 2021 (Table 1).

### 2.1. Overall AMU Per Farm and Age Group

**Figure 1:**
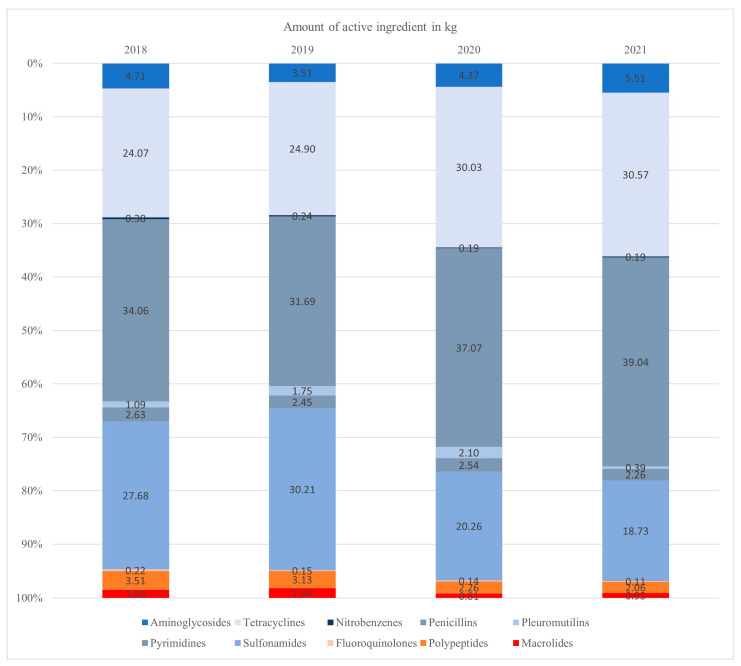
The relative distribution of antimicrobial usage between different antimicrobial classes was assessed based on the amount of active ingredient used. The antimicrobial usage of third- and fourth-generation cephalosporines, clavulanates and lincosamides is not represented due to their low usage levels. Highest Priority Critically Important Antimicrobial classes are shown in reddish colors at the bottom of each bar.

The cumulative AMU per farm from 2018 to 2021 was assessed using different measures, including the active ingredient, ATI, TIADD, and TIUDD The results are presented in Table 2 and Table 3 and Figure 1.

The largest quantity of active ingredient was given to the age group of fattening pigs in all the years studied. The suckling piglets received the smallest amount of active ingredient.

In summary, the analysis showed a significant decrease in active ingredient consumption in kilograms in 2021 compared to all the previous years, while there was a significant increase in 2021 based on the indicators TIADD and TIUDD, respectively, and a significant increase between 2021 and 2018 for the ATI indicator.

### 2.2. AMU Per Age Category

Regarding the age category of suckling piglets, a significant decrease in active ingredient consumption in 2021 compared to 2018 and 2019 (likewise between 2020 and 2019) was observed. (Supplementary Material Table S1). No statistically significant difference was observed between the years 2018 and 2021 for ATI, TIADD and TIUDD in the suckling piglets.

The age category of weaners showed a significant decrease in active ingredients in 2021 compared to 2019, but no other significant changes based on any other indicator were observed.

The analysis showed no significant changes in the amount of active ingredients in fattening pigs. In contrast, all other indicators showed a stepwise increase in AMU year after year, which was significant for the comparison between 2021 and all other years for ATI, TIADD and TIUDD, respectively. In addition, the comparison of 2020 to all other years for ATI and TIUDD, as well as the comparison of 2020 to 2018 for TIADD, was also significant.

In gestating sows, a significant increase in AMU based on active ingredients was noted in the year 2019. Furthermore, when assessing ATI, TIADD and TIUDD, a significant increase was observed in 2021 and 2020 in comparison to 2018.

No significant change in AMU based on active ingredients was observed among lactating sows. Conversely, a significant increase was noted in the ATI and TIUDD in the years 2021 and 2020, relative to 2018. Furthermore, the TIADD also showed a significant rise in all years compared to 2018.

### 2.3. AMU Quantification Per Antimicrobial Classes

The active ingredient quantities for different classes of antimicrobials are aggregated and presented in Table 4. The distribution of these quantities is graphically displayed in Figure 1.

Based on the amount of active ingredient used, the largest proportion of AMU was attributed to penicillins each year, followed by sulfonamides and tetracyclines. Between 2018 and 2019, the consumption of sulfonamides was higher than the consumption of tetracyclines. However, in the years 2020 and 2021, the consumption of tetracyclines exceeded that of sulfonamides. Other than sulfonamides, the antimicrobial classes of tetracyclines and penicillin did not exceed 15% in total.

**Figure 2:**
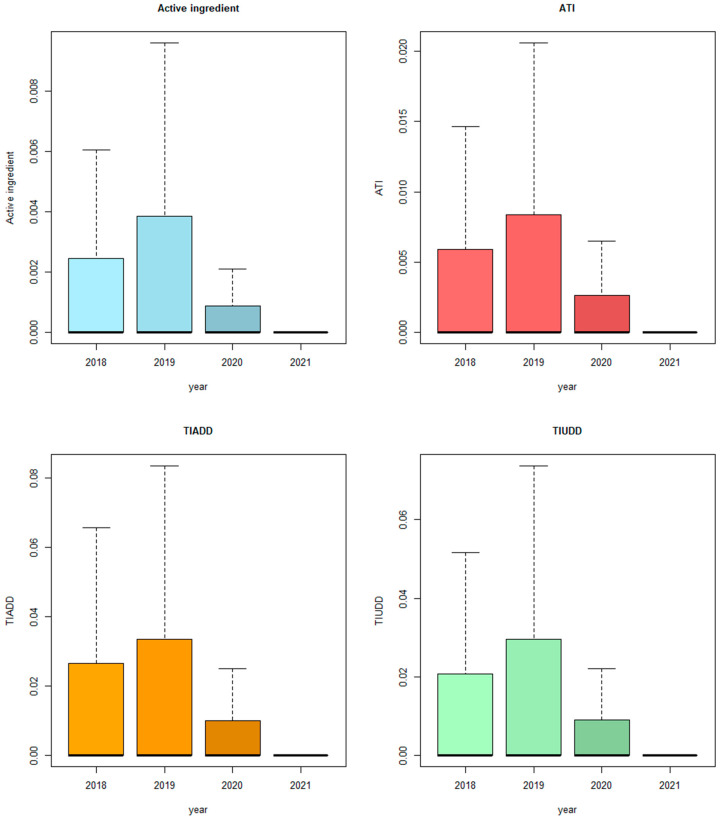
Overall comparison of antimicrobial usage of the Highest Priority Critically Important Antimicrobial classes between the years 2018 and 2021, assessed through the active ingredient in kilograms, treatment days per farm (ATI) and treatment incidence based on defined daily doses (TIADD) or used daily doses (TIUDD).

The AMU of third- and fourth-generation cephalosporines, clavulanates and lincosamides is not represented in Figure 2 due to their low usage levels (highest usage level < 0.11% per year).

### 2.4. HPCIAs

The relative amount of HPCIAs compared to the total AMU measured in kilograms decreased continuously over the years (Figure 1). The class of HPCIAs most frequently used was polypeptides. Within the overall usage, HPCIAs accounted for 5.23% in 2018, 5.12% in 2019, 3.21% in 2020 and 3.10% in 2021.

Looking at the overall usage of HPCIAs, a significant decrease was observed in 2020 and 2021 compared to the year 2019, in which the highest usage was shown. This observation was made regardless of the active ingredient, and whether ATI, TIADD or TIUDD were used as AMU measurement method. Furthermore, a significant reduction between 2021 and 2020 was observed for AMU based on each indicator (Figure 2). Like the overall results, the AMU of HPCIAs shows a peak in 2019 and, in comparison, a decreased usage in 2021 for the age category of suckling piglets that was significant for active ingredient, ATI and TIUDD, respectively.

When analyzing the AMU of HPCIAs in weaned piglets, a significant reduction was observed in the year 2021 when compared to 2019 and 2018, as well, regardless of which indicator was used. For the group of fattening pigs, a significant decrease in active ingredient consumption was observed in both 2021 and 2020 when compared to 2019. In contrast, AMU based on ATI, TIADD and TIUDD showed an increase in 2021, which was significant compared to the year 2019. Upon analysis of antimicrobial consumption in gestating sows, it was found that there was a significant reduction in the active ingredient consumption, ATI, TIADD and TIUDD in 2021 compared to 2018 and 2019. In lactating sows, significant decreases in 2021 compared to 2019 were observed in active ingredient, ATI, TIADD and TIUDD (Supplementary Material Table S2).

### 2.5. Interventions

No intervention data from 2018 was included in the analysis. Interventions implemented in 2019 resulted in a decrease in AMU at four farms, but an increase in AMU was observed at four other farms in 2020. The interventions had no effect on the AMU for eight farms. In 2020, 207 interventions were carried out in total, which led to a reduction in AMU at 105 farms, while an increase in AMU was observed at 39 farms. No change was observed at 63 farms.

## 3. Discussion

### 3.1. Overall AMU

#### 3.1.1. Sample Size

A persistent increase in the overall number of farms, the number of farms using antimicrobials and the number of pigs per age category was observed. A possible justification for the substantial surge in the number of farms could be attributed to the compulsory implementation of the SuisSano/Safety + Health program in April 2020. Over the course of the years studied, more and more farms have participated in the SuisSano/Safety + Health program, resulting in 3539 out of a total of 5561 farms registered in Switzerland in 2021, which corresponds to a coverage of 60% [15]. For 2021, over 2 million fattening pigs were included in the analysis, which corresponds to coverage of over 80% of the total pig population in Switzerland from the same year [15]. A comparison of the years 2018 and 2021 shows that the largest relative increase in the study population occurred in fattening pigs. The study included breeding farms, fattening farms and closed farms. It can be concluded that relatively more fattening farms joined the program over the years. This could be related to the fact that the program was voluntary before 2020, and perhaps more specialized breeding farms participated at the beginning of the program, while the fattening farms joined later, after 2020, and during the observation period shown in this study.

#### 3.1.2. Calculation Methods

This study analyzed the results of four different calculation methods, each with advantages and disadvantages. For example, data on the active ingredient and ATI are easy to collect but do not include the dosage of various active ingredients and can therefore lead to misinterpretations or failure to recognize incorrect treatments. In contrast, TIADD and TIUDD, as treatment equivalents that include dosage information, are better suited for monitoring a population if the data are correctly sampled and broadly covered. Each method therefore has its justification, depending on the data situation and the desired depth of analysis.

#### 3.1.3. AMU between Years

The AMU, measured in kilograms per farm, increased from 2018 to 2019 and then decreased, which might be linked to a shift to the usage of more potent antimicrobial classes in 2020 and 2021. However, the AMU measured in ATI, TIADD and TIUDD increased from 2018 to 2021. One plausible explanation for the observed increase in AMU equivalents measured by this treatment could be that the mandatory implementation of the SuisSano Safety + program led to the inclusion of less-motivated farms, resulting in the incorporation of high-consumption farms. Notably, fattening pigs account for the observed increase in 2021 across all indicators, which may be linked to a lack of specialization on fattening farms newly included in the program and lower awareness of prudent usage of antimicrobials. Another conceivable factor that may have contributed to this trend is the age distribution of animals on the farm, as well as the number of animals per farm, as younger animals and larger farms might have a higher AMU. According to a study conducted in Switzerland, a notable proportion of antimicrobial treatments (94%) were administered to suckling piglets and weaners. Moreover, the study found that an amplified herd size is positively associated with an increase in antimicrobial use [16]. In contrast, in Belgium, larger herds tend to use fewer antimicrobials due to better biosecurity measures [17]. Furthermore, a German paper has demonstrated an association between AMU and factors such as herd size and farm organization. The authors of the German study also reported that the highest quantities of antimicrobials were prescribed to fattening pigs [18]. In several other studies, including a Swiss one, weaned pigs are the age group that received the most treatments [19,20,21]. This contrasts with the result in our study, where the fattening pigs receive the most active ingredient. Further investigations would be necessary to identify the underlying causes of these disparities, but a direct comparison is only valid with the same measurement methodology, and the time course must also be considered, as the study presented here observed a significant decrease in AMU for weaners in 2021. One possible explanation for the significant reduction could be the use of more potent preparations with a higher therapeutic efficacy [22]. Based on our findings, this theory gains support as we observed a reduction in AMU in kilograms, particularly in fattening pigs and sows. However, when analyzing the indicators of ATI, TIADD and TIUDD, an upward trend becomes apparent. It is crucial to acknowledge that not all countries can collect such a wide range of data, which opens the possibility of concealed increases or feigned decreases. It is imperative to account for this aspect when conducting country comparisons based on kg/PCU.

In gestating sows, a significant increase in the amount of active ingredients was observed in 2019 compared to 2018 but not in the other indicators. This observation could be indicative of the utilization of older generations of antibiotics, as they typically require higher therapeutic dosages when compared to newer generations. Conversely, an increase in ATI in 2020 and 2021 compared to 2018 suggests that less-motivated piglet-producing farms may have joined the program in 2019 for financial reasons since farmers were paid for joining the SuisSano Safety + program before it became mandatory in 2020.

According to the European Medicines Agency (EMA), Switzerland held a moderate position in terms of antimicrobial agent sales data in 2017. However, the country has now achieved a more favorable international position in terms of its antimicrobial sales data [23]. Nevertheless, given the high health status of Swiss pigs, one might anticipate a lesser amount of AMU [24]. However, the Swiss Antibiotic Resistance Report (SARR) of 2022 reveals that the use of antimicrobial agents in veterinary medicine in Switzerland has consistently declined over time, with sales data decreasing at a faster rate than the corresponding biomass, without separating this decrease between different species [9].

### 3.2. Antimicrobial Classes and HPCIAs

The majority of AMU in each year was primarily attributed to penicillins, followed by sulfonamides and tetracyclines, based on the quantity of the active ingredient in kilograms used. According to the SARR of 2022, in the porcine population, the most frequently utilized class of antimicrobials were tetracyclines (32.5%), sulfonamides (28.0%) and penicillins (25.1%). The HPCIAs most frequently used were macrolides while, in our study, it was polypeptides [7]. However, it should be noted that the SARR is based on sales data, while our investigation relied on consumption data. In a comparative study among Germany, Sweden, Belgium and France, it was observed that penicillins were the most used antimicrobial class in Belgium, Germany and Sweden, while polypeptides were the most frequently used in France [25]. In another more recent study in Germany, penicillins were also identified as the most used class of antimicrobials [26]. In contrast to the observed prevalence of penicillins as the most used antimicrobial class in many European countries, tetracyclines were reported to be the most frequently used antimicrobial class in countries such as Japan and the USA [27,28].

As for the HPCIAs, a clear trend could be observed in our research: The amount of HPCIAs used reduced over the years. A possible factor in this clear reduction could be the introduction of steering factors into the program. Each use of an HPCIA is punished with a factor four in the program. Similar results were observed in Denmark, where steering factors led to a reduction in AMU [12,13]. However, since not only the total amount but also the ATI, TIADD and TIUDD were reduced in our study, there seems to be an actual reduction here.

Both in total and relative terms, polypeptides corresponding to the active substance colistin represent the largest group within the HPCIAs, so this antimicrobial class may have the highest potential for further reduction. This is particularly important in view of the fact that mobilized colistin resistance genes (*mcr1* and *mcr2*) have also been found in pigs in Switzerland, although resistance to colistin from *Escherichia coli* isolated from pig’s intestine was still of low prevalence in an earlier study [29,30].

### 3.3. Interventions

Interventions taken in 2019 showed less effect than interventions taken in 2020 where, in 50.72% of the farms, the AMU measured in ATI was reduced. Still, the other half of the farms showed no change or even an increase in AMU. Therefore, a general statement on whether the intervention was a success in relation to AMU is difficult. Another study looking at the impact of the SuisSano/Safety + Health program per se showed no significant difference in AMU per farm through the induction of the program alone. On the other hand, a study conducted in Switzerland on postpartum dysgalactia found that a close herd management approach can result in a notable reduction in antimicrobial use [31]. As in the current study, data about the kind and interval of intervention is missing, so a detailed conclusion as to why intervention based on AMU within the program succeeded on some farms while failing on other ones is not possible. Other factors such as the number of sows present at the site, biosecurity measurements and location of the farm may also play important roles in AMU [32].

### 3.4. Limitations

The accuracy and quality of the data may depend on the education and attention to detail of the person entering it, potentially leading to deviations in the results.

The authors did not influence the selection of the farms. However, it is plausible that farms with higher motivation to reduce their antimicrobial use participated voluntarily at the beginning of the program. Conversely, less motivated farms with higher antimicrobial consumption may have joined the program only after participation became mandatory. This may have introduced bias into the data.

## 4. Materials and Methods

### 4.1. Data Collection

The study involved all the farms participating in the SuisSano/Safety + Health program, which started in 2015. In 2018, the treatment journal was implemented in an electronic form. The data analyzed spanned four years from 2018 to 2021. Participating farms are obligated to report all on-farm medication usage, including AMU. The start and end dates of treatment, age group (such as suckling piglets, weaners, fattening pigs, gilts, lactating sows, and gestating sows including boars), corresponding animal weights, number of applications, affected organ systems, health issues, preparation names and the amount of medication used are mandatory treatment data within the ETJ. Additionally, farm-level data, such as the number of animals in each age category, are integrated into the program. These data are used as the number of pigs at risk, and either the total number of pigs housed per year (piglets, weaners, fattening pigs) or the average number of housed pigs per year (lactating sows and gestating sows) were included as the denominator in the calculations. The weight of the animals is estimated by the farmer. All gilts weighing between 25 and 140 kg were considered fatteners, while gilts weighing less than 25 kg or more than 140 kg were excluded from the analysis. Furthermore, records with missing information were not included in the analysis.

### 4.2. Quantification of AMU

The data was exported as a CSV file and imported into Microsoft Excel 365 version 2402.

The AMU was calculated as the amount of active ingredient in kilograms, as treatment days per farm (ATI) and treatment incidence (TI), based either on animal-defined daily doses (TIADD) or used daily doses (TIUDD). The amount of active ingredient of each treatment is given by the specific concentration and dosage of each preparation used on the farm. The indicator ATI was defined as published by Blaha et al., 2006 [33], and calculated as follows:

$$\frac{number\;of\;animals\times number\;of\;treatment\;days}{number\;of\;pigs\;at\;risk}$$

The ATI was defined as the indicator that is reported by the farms within the SuisSano/Safety + Health program. Before reporting, the usage of HPCIAs is multiplied by a steering factor of four to control these antimicrobial classes. The factors are not included in our analysis. The TI was calculated as the TIADD, and the TIUDD was based on the formula by Timmerman et al. (2006) [34].
**TIADD_pig_**$\frac{Total\;amount\;of\;an\;antimicrobial\;drug\;used(mg)}{ADDpig\left(\frac{mg}{kg}\right)\times observation\;period\left(days\right)\times kg\;pigs\;at\;risk}\times 1000\;pigs\;at\;risk$**TIUDD_pig_**$\frac{Total\;amount\;of\;anantimicrobial\;drug\;used(mg)}{UDDpig\left(\frac{mg}{kg}\right)\times observation\;period\left(days\right)\times kg\;pigs\;at\;risk}\times 1000\;pigs\;at\;risk$

The TIADD is based on the nationally defined daily doses for each individual antimicrobial preparation that can be administered to pigs in Switzerland [35]. The TIUDD is based on the exact daily dose used by the farmer for each individual treatment and is considered the most accurate dosage within the different monitoring calculations. By using both indicators simultaneously, it is possible to highlight the difference between a dosage that is accepted by the authorities and the dosage that is actually used to treat the pigs. The difference is also an indication of the compliance within the population of farms. The observation period was defined according to the average time each age group spends on the Swiss farm: for suckling pigs—30 days, for weaners—41 days, for fattening pigs—101 days and for sows (lactating and pregnant)—365 days.

Long-acting formulations vary in their duration of action depending on, e.g., the active ingredient used or the concentration within a preparation. In addition, antimicrobial manufacturers do not always provide information on the duration of action, which is needed to calculate the treatment duration needed for this study [36]. Consequently, correction factors for long-acting formulations were not considered within the calculations. Therefore, the results of the study are more comparable to results from other countries [36].

### 4.3. Data Analysis

The data were analyzed with Microsoft Excel 365, and the statistical analysis was conducted by RStudio Team (2020 RStudio: Integrated Development for R. RStudio, PBC, Boston, MA, USA, http://www.rstudio.com/, accessed on 1 March 2024). All the datasets were checked for normality by a histogram followed by the Shapiro–Wilk test for normality. Depending on the normality, the ANOVA or Kruskal–Wallis test was chosen to compare the AMU of the different years based on the indicators described. Post hoc analysis adjusted after Bonferroni correction was used to show significant differences between certain years. *p*-values below 0.05 were set to indicate significance.

### 4.4. Interventions

The implementation of interventions such as on-farm visits, phone calls, or notes were carried out by the Swiss pig health services or veterinarians with specialized training. These interventions were exclusively provided to farms with high AMU usage in a certain year, which were identified based on a rolling benchmarking system. Each age group was assessed separately. The authors were provided with an anonymized list of farms indicating the type of intervention implemented. Given the lack of age group information and the possibility of multiple interventions per year, only the effect of interventions on mean ATI progression was analyzed. The year 2018 was excluded from the analysis since only two farms received an intervention that year. The criterion for defining an increase or decrease in AMU was based on a change greater than 20%.

## 5. Conclusions

This study shows a reduction in the total amount of antimicrobials by monitoring the AMU in Swiss pigs from 2019 to 2021 but, during the same period, more treatment equivalents measured as treatment days or treatment incidence per farm were observed. Looking specifically at the usage of HPCIAs, a reduction—regardless of which indicator was used—was observed during the observation period. Only in fattening pigs did the number of treatments per farm with HPCIAs increase, showing the importance of the age category for further reduction. Through the electronic treatment journal, a nationwide standardized analysis of AMU became possible for the first time, although weaknesses due to digital recording by individual farmers must be taken into account, as the analyses can only be as good as the individual data entries. The firstly voluntary and, since 2020, mandatory monitoring program in pig production in Switzerland included farms with the potential for AMU reduction. Besides the current intervention in high-usage farms, other individual factors like herd size, housing, feeding, management, biosecurity, interval and the kind of intervention should be taken into account to combine the AMU with the health status of the animals in a meaningful way. It is crucial to prioritize animal welfare when considering antimicrobial use. If an animal is ill and requires antimicrobials to alleviate suffering and promote recovery, it is necessary to provide appropriate treatment. However, it is also essential to use antimicrobials judiciously and implement measures to prevent the unnecessary use of antimicrobials, which can contribute to the development of antimicrobial resistance and compromise animal and human health in the long term.

## Figures and Tables

**Table 1:** The number of registered pigs by age group and farms in the electronic treatment journal per year (2018–2021). The numbers of farms using antimicrobials are given in brackets.

Age Group	2018	2019	2020	2021
Suckling piglets (*n*)	1’317’592	1’584’680	1’912’505	2’327’035
Weaners (*n*)	1’132’360	1’361’430	1’639’831	1’996’143
Fattening pigs (*n*)	858’294	1’095’041	1’606’793	2’032’961
Lactating sows (*n*)	14’762	17’869	22’900	28’259
Gestating sows (*n*)	38’437	46’060	58’984	71’638
Farms (*n*)	1213 (1160)	1526 (1480)	2339 (2225)	3539 (3288)

**Table 2:** Summary of overall antimicrobial usage from 2018 to 2021 in kilograms of active ingredient per age group.

Year	Suckling Piglet	Weaner	Fattening Pig	Gestating Sow	Lactating Sow
2018	28.69	236.25	292.38	80.46	101.59
2019	43.14	311.29	564.98	142.18	141.62
2020	50.88	297.87	666.04	205.61	208.28
2021	66.19	371.74	891.79	300.77	240.05
Sum	188.89	1217.14	2415.19	729.03	691.54

**Table 3:** Summary of overall antimicrobial usage from the years 2018 to 2021 based on the four different measuring methods (the active ingredient in kilograms, treatment days per farm (ATI) and treatment incidence based on defined daily doses (TIADD) or used daily doses (TIUDD)). Significant differences between the years are indicated by differences between the letters. A combination of letters does not significantly differ from the corresponding single-letter groups.

Overall AMU Per Farm		Year	Mean (95% Conf.)	Median	SD	Min	Max	Significance
	Active ingredient							
		2018	0.64 (0.49–0.78)	0.15	2.52	0	61.05	A
		2019	0.81 (0.66–0.97)	0.18	3.01	0	84.06	A
		2020	0.64 (0.57–0.72)	0.15	1.82	0	45.31	A
		2021	0.57 (0.5–0.64)	0.11	2.11	0	71.23	B
	ATI							
		2018	0.05 (0.04–0.06)	0.02	0.18	0	4.17	A
		2019	0.06 (0.05–0.07)	0.02	0.20	0	3.36	AB
		2020	0.07 (0.06–0.07)	0.02	0.23	0	4.67	AB
		2021	0.09 (0.08–0.11)	0.02	0.42	0	11.00	B
	TIADD							
		2018	0.25 (0.18–0.33)	0.08	1.28	0.	27.72	A
		2019	0.33 (0.25–0.4)	0.08	1.55	0	28.22	AB
		2020	0.46 (0.35–0.57)	0.09	2.54	0	80.40	B
		2021	0.77 (0.62–0.92)	0.11	4.43	0	108.91	C
	TIUDD							
		2018	0.27 (0.17–0.32)	0,07	1.75	0.	11.25	A
		2019	0.32 (0.24–0.4)	0.07	1.65	0.	28.38	AB
		2020	0.43 (0.34–0.52)	0.08	2.20	0.	46.21	B
		2021	0.72 (0.58–0.86)	0.09	4.10	0	108.91	C

**Table 4:** Antimicrobial usage measured as active ingredients in kilograms in the years 2018 to 2021, separated by the different antimicrobial classes. The Highest Priority Critically Important Antimicrobial classes are highlighted in red.

Antimicrobial Classes	2018	2019	2020	2021
Aminoglycosides	34.83	42.26	62.47	103.01
Cephalosporins 3. Generation	0.03	0.04	0.03	0.03
Cephalosporins 4. Generation	0.13	0.15	0.13	0.14
Clavulanates	0.47	0.61	1.64	2.31
Fluoroquinolones	1.66	1.84	2.06	1.97
Lincosamides	0.51	0.86	1.43	1.54
Macrolides	11.03	22.12	11.57	17.36
Nitrobenzoles	2.77	2.86	2.67	3.52
Penicillins	251.85	381.24	529.57	730.33
Pleuromutilins	8.04	21.05	30.06	7.29
Polypeptides	25.97	37.68	32.30	38.57
Pyrimidines	19.48	29.47	36.30	42.31
Sulfonamides	204.66	363.48	289.48	350.29
Tetracyclines	177.93	299.55	428.97	571.88
Total	739.37	1203.20	1428.67	1870.55

## Data Availability

The data that support the findings of this study are available on request from the corresponding author (T.E.).

## Supplementary Materials

The following supporting information can be downloaded at: https://www.mdpi.com/article/10.3390/antibiotics13090831/s1, Table S1: Summarizes the overall (AMU) from 2018 to 2021, Table S2: Summarizes the AMU of HPCIAs from 2018 to 2021.
