# Supplementary material for: Trends in Antimicrobial Usage on Swiss Pig Farms from 2018 to 2021: Based on an Electronic Treatment Journal
Source: Antibiotics (Basel). 2024 Sep 2;13(9):831. doi: 10.3390/antibiotics13090831 (PMC11440108; doi:10.3390/antibiotics13090831)
Supplement: Supplementary file 1 [file antibiotics-13-00831-s001.zip › antibiotics-3113695-supplementary.pdf]

Supplementary material

Table S1 summarizes the overall (AMU) from 2018 to 2021. Significance is indicated by complete differences between the letters. A combination of letters does not significantly differ from the corresponding single letter groups.

**AMU per farm in suckling piglets**		**Year**	**Mean (95% Conf.)**	**Median**	**SD**	**Min**	**Max**	**Significance**
	Active ingredient							
		2018	0.05 (0.04-0.06)	0.01	0.11	0	1.13	AB
		2019	0 06 (0.05-0.07)	0.01	0.17	0	2.11	A
		2020	0.05 (0.04-0.06)	0.01	0.15	0	2.31	BC
		2021	0.05 (0.04-0.06)	0.01	0.19	0	5.09	C
	TBI							
		2018	0.02 (0.01-0.02)	0.01	0.04	0	0.47	A
		2019	0.02 (0.01-0.02)	0.01	0.03	0	0.41	A
		2020	0.02 (0.01-0.02)	0.01	0.04	0	0.54	A
		2021	0.02 (0.01-0.02)	0.01	0.03	0	0.41	A
	TI_{ADD}							
		2018	0.09 (0.07-0.12)	0.03	0.36	0	7.59	A
		2019	0.08 (0.07-0.09)	0.03	0.17	0	1.98	A
		2020	0.10 (0.08-0.13)	0.03	0.47	0	10.82	A
		2021	0.08 (0.07-0.09)	0.03	0.17	0	2.65	A
	TI_{UDD}							
		2018	0.05 (0.03-0.06)	0.02	0.10	0	1.29	A
		2019	0.04 (0.03-0.05)	0.02	0.08	0	1.13	A
		2020	0.05 (0.04-0.05)	0.02	0.10	0	1.48	A
		2021	0.04 (0.04-0.05)	0.02	0.09	0	1.13	A

**AMU per farm in weaners**		**Year**	**Mean (95% Conf.)**	**Median**	**SD**	**Min**	**Max**	**Significance**
	Active ingredient							
		2018	0.37 (0.23-0.51)	0.01	1.79	0	25.26	A
		2019	0 40 (0.27-0.53)	0.01	1.85	0	30.61	A
		2020	0.28 (0.22-0.34)	0.01	0.99	0	14.29	AB
		2021	0 26 (0.20-0.33)	0.01	1.15	0	20.30	B
	TBI							
		2018	0.05 (0.04-0.06)	0.00	0.16	0	2.11	A
		2019	0.06 (0.05-0.07)	0.00	0.16	0	1.75	A
		2020	0.06 (0.05-0.08)	0.00	0.18	0	1.71	A
		2021	0.06 (0.05-0.07)	0.00	0.20	0	2.91	A
	TI_{ADD}							
		2018	0.17 (0.12-0.22)	0.01	0.62	0	10.64	A
		2019	0.17 (0.13-0.20)	0.01	0.49	0	5.62	A
		2020	0.19 (0.16-0.23)	0.01	0.58	0	10.33	A
		2021	0.18 (0.15-0.21)	0.01	0.59	0	9.23	A
	TI_{UDD}							
		2018	0.14 (0.11-0.18)	0.01	0.44	0	5.78	A
		2019	0.17 (0.13-0.19)	0.01	0.45	0	4.80	A
		2020	0.18 (0.15-0.21)	0.01	0.49	0	4.70	A
		2021	0.17 (0.15-0.20)	0.01	0.54	0	7.96	A

**AMU per farm in fattening pigs**		**Year**	**Mean (95% Conf.)**	**Median**	**SD**	**Min**	**Max**	**Significance**
	Active ingredient							
		2018	0.29 (0.16-0.43)	0.02	2.20	0	61.05	A
		2019	0 44 (0.29-0.60)	0.02	2 80	0	84.06	A
		2020	0 34 (0.27-0.41)	0.02	1.59	0	45 31	A
		2021	0.30 (0.24-0.36)	0.02	1.69	0	71.23	A
	TBI							
		2018	0.03 (0.02-0.05)	0.00	0.20	0	4.17	A
		2019	0.04 (0.03-0.05)	0.00	0.22	0	3.25	A
		2020	0.05 (0.04-0.06)	0.00	0.25	0	4.67	B
		2021	0.08 (0.07-0.10)	0.00	0.44	0	11.00	C
	TI_{ADD}							
		2018	0.28 (0.18-0.37)	0.03	1.48	0	27.72	A
		2019	0.41 (0.30-0.53)	0.03	2.11	0	33.21	AB
		2020	0.53 (0.40-0.65)	0.03	2.80	0	80.40	B
		2021	0.86 (0.69-1.03)	0.05	4.70	0	108.91	C
	TI_{UDD}							
		2018	0.30 (0.18-0.42)	0.02	1.95	0	41.25	A
		2019	0.39 (0.27-0.50)	0.02	2.15	0	32.18	A
		2020	0.49 (0.39-0.60)	0.03	2.43	0	46.21	B
		2021	0.80 (0.64-0.95)	0.04	4.32	0	108.91	C

**AMU per farm in gestating sows**		**Year**	**Mean (95% Conf.)**	**Median**	**SD**	**Min**	**Max**	**Significance**
	Active ingredient							
		2018	0.17 (0.14-0.20)	0.05	0.35	0	3.10	A
		2019	0.27 (0.21-0.33)	0.09	0.77	0	12.09	B
		2020	0.24 (0.19-0.29)	0.06	0.78	0	12.19	A
		2021	0 26 (0.17-0.34)	0.05	1.49	0	47.51	A
	TBI							
		2018	0.04-0.05)	0.03	0.05	0	0.65	A
		2019	0.08 (0.03-0.14)	0.03	0.71	0	16.67	AB
		2020	0.06 (0.05-0.07)	0.03	0.11	0	1.83	B
		2021	0.06 (0.05-0.06)	0.03	0.09	0	1.50	B

	TI_{ADD}							
		2018	0.11 (0.10-10.13)	0.08	0.15	0	1.94	A
		2019	0.24 (0.07-0.41)	0.09	2.06	0	48.71	AB
		2020	0.18 (0.15-0.20)	0.10	0.36	0	5.23	B
		2021	0.18 (0.16-0.19)	0.10	0.29	0	4.93	B
	TI_{UDD}							
		2018	0.11 (0.10-0.12)	0.08	0.14	0	1.78	A
		2019	0.23 (0.07-0.39)	0.08	1.93	0	45.66	AB
		2020	0.16 (0.14-0.18)	0.09	0.30	0	5.02	B
		2021	0 16 (0.15-0.18)	0.09	0.25	0	4.11	B

**AMU per farm in lactating sows**		**Year**	**Mean (95% Conf.)**	**Median**	**SD**	**Min**	**Max**	**Significance**
	Active ingredient							
		2018	0.16 (0.14-0.18)	0.07	0.28	0	2.58	A
		2019	0.19 (0.16-0.21)	0.09	0.31	0	2.88	A
		2020	0.20 (0.18-0.22)	0.08	0.34	0	3.77	A
		2021	0.17 (0.16-0.19)	0.08	0.33	0	5.93	A
	TBI							
		2018	0.10 (0.10-0.11)	0.08	0.10	0	0.95	A
		2019	0.12 (0.11-0.13)	0.09	0.18	0	1.45	AB
		2020	0.13 (0.12-0.13)	0.10	0.12	0	1.00	B
		2021	0.14 (0.13-0.15)	0.10	0.16	0	2.17	B
	TI_{ADD}							
		2018	0.29 (0.27-0.31)	0.23	0.29	0	2.92	A
		2019	0.35 (0.32-0.37)	0.25	0.39	0	4.04	B
		2020	0.37 (0.34-0.39)	0.27	0.39	0	5.22	B
		2021	0.41 (0.38-0.44)	0.28	0.54	0	6.85	B
	TI_{UDD}							
		2018	0.29 (0.26-0.31)	0.23	0.27	0	2.60	A
		2019	0.34 (0.31-0.36)	0.25	0.35	0	3.97	AB
		2020	0.34 (0.33-0.36)	0.27	0.34	0	2.74	B
		2021	0.38 (0.36-0.41)	0.27	0.45	0	5.94	B

Table S2 summarizes the AMU of HPCIAs from 2018 to 2021. Significance is indicated by complete differences between the letters. A combination of letters does not significantly differ from the corresponding single letter groups.

AMU per farm in suckling piglets		Year	Mean (95% Conf.)	Median	SD	Min	Max	Significance
	Active ingredient							
		2018	0.00 (0.00- 0.00)	0	0.01	0	0.13	A
		2019	0.00 (0.00- 0.00)	0	0.01	0	0.24	A
		2020	0.00 (0.00- 0.00)	0	0.01	0	0.29	AB
		2021	0.00 (0.00- 0.00)	0	0.01	0	0.13	B
	TBI							
		2018	0.01 (0.01-0.01)	0	0.02	0	0.22	AB
		2019	0.01 (0.01-0.01)	0	0.05	0	0.80	A
		2020	0.01 (0.01-0.02)	0	0.11	0	2.92	AB
		2021	0.01 (0.01-0.01)	0	0.03	0	0.42	B
	TI_{ADD}							
		2018	0.03 (0.02-0.03)	0	0.09	0	1.21	A
		2019	0.04 (0.03-0.05)	0	0.16	0	2.32	A
		2020	0.04 (0.03-0.06)	0	0.30	0	8.00	A
		2021	0.02 (0.02-0.03)	0	0.09	0	1.16	A
	TI_{UDD}							
		2018	0.02 (0.02-0.02)	0	0.06	0	0.59	AB
		2019	0.03 (0.02-0.04)	0	0.14	0	2.19	A
		2020	0.04 (0.02-0.05)	0	0.30	0	8.00	AB
		2021	0.02 (0.02-0.02)	0	0.08	0	1.16	B

AMU per farm in weaners		Year	Mean (95% Conf.)	Median	SD	Min	Max	Significance
	Active ingredient							
		2018	0.05 (0.03-0.06)	0	0.20	0	3.27	A
		2019	0.05 (0.03-0.07)	0	0.26	0	4.38	A
		2020	0.03 (0.02-0.04)	0	0.14	0	3.32	AB
		2021	0.03 (0.02-0.03)	0	0.13	0	2.56	B
	TBI							
		2018	0.07 (0.05-0.08)	0	0.19	0	2.11	A
		2019	0.07 0.06-0.08)	0	0.19	0	1.75	A
		2020	0.08 (0.07-0.10)	0	0.25	0	2.40	AB
		2021	0.07 (0.06-0.08)	0	0.20	0	2.00	B
	TI_{ADD}							
		2018	0.20 (0.15-0.25)	0	0.66	0	10.32	A
		2019	0.20 (0.16-24)	0	0.60	0	6.59	A
		2020	0.24 (20-0.28)	0	0.71	0	6.58	AB
		2021	0.19 (0.16-0.22)	0	0.58	0	5.69	B
	TI_{UDD}							
		2018	0.18 (0.14-0.22)	0	0.51	0	5.78	A
		2019	0.19 (0.16-0.23)	0	0.52	0	4.80	A
		2020	0.23 (0.19-0.27)	0	0.67	0	6.58	AB
		2021	0.18 (0.15-0.21)	0	0.55	0	5.48	B

AMU per farm in fattening pigs		Year	Mean (95% Conf.)	Median	SD	Min	Max	Significance
	Active ingredient							
		2018	0.01 (0.00-0.01)	0	0.10	0	3.00	AB
		2019	0.01 (0.01-0.02)	0	0.15	0	4.54	A
		2020	0.00 (0.00-0.01)	0	0.04	0	1.26	B
		2021	0.01 (0.00-0.01)	0	0.05	0	1.08	B
	TBI							
		2018	0.01 (0.01-0.02)	0	0.14	0	2.80	AB
		2019	0.03 (0.02-0.04)	0	0.22	0	3.33	A
		2020	0.02 (0.01-0.03)	0	0.17	0	3.00	B
		2021	0.03 (0.02-0.04)	0	0.29	0	11	B
	TI_{ADD}							
		2018	0.13 (0.05-0.22)	0	1.37	0	27.49	AB
		2019	0.27 (0.16-0.38)	0	2.01	0	31.73	A
		2020	0.20 (0.12-0.27)	0	1.67	0	26.73	B
		2021	0.31 (0.21-0.42)	0	3.05	0	108.91	B
	TI_{UDD}							
		2018	0.13 (0.05-0.22)	0	1.36	0	27.72	AB
		2019	0.28 (0.16-0.40)	0	2.15	0	33.00	A
		2020	0.20 (0.12-0.28)	0	1.69	0	29.70	B
		2021	0.30 (0.20-0.41)	0	2.89	0	108.91	B

AMU per farm in gestating sows		Year	Mean (95% Conf.)	Median	SD	Min	Max	Significance
	Active ingredient							
		2018	0.00 (0.00-0.00)	0	0.01	0	0.15	A
		2019	0.00 (0.00-0.00)	0	0.01	0	0.10	A
		2020	0.00 (0.00-0.00)	0	0.00	0	0.03	AB
		2021	0.00 (0.00-0.00)	0	0.00	0	0.04	B
	TBI							
		2018	0.01 (0.00-0.01))	0	0.03	0	0.28	A
		2019	0.00 (0.00-0.01))	0	0.02	0	0.28	A
		2020	0.00 (0.00-0.00)	0	0.02	0	0.42	AB
		2021	0.00 (0.00-0.00)	0	0.02	0	0.18	B
	TI_{ADD}							
		2018	0.02 (0.01-0.02)	0	0.08	0	0.83	A
		2019	0.01 (0.01-0.02)	0	0.07	0	1.52	A

		Year	Mean (95% Conf.)	Median	SD	Min	Max	Significance
		2020	0.01 (0.01-0.01)	0	0.06	0	1.16	AB
		2021	0.01 (0.01-0.01)	0	0.04	0	0.57	B
	TI_{UDD}							
		2018	0.02 (0.01-0.02)	0	0.07	0	0.76	A
		2019	0.01 (0.01-0.01)	0	0.05	0	0.76	A
		2020	0.01 (0.00-0.01)	0	0.05	0	1.16	AB
		2021	0.01 (0.01-0.01)	0	0.04	0	0.48	B

**AMU per farm in lactating sows**		**Year**	**Mean (95% Conf.)**	**Median**	**SD**	**Min**	**Max**	**Significance**
	Active ingredient							
		2018	0.00 (0.00-0.00)	0	0.01	0	0.07	AB
		2019	0.00 (0.00-0.00)	0	0.01	0	0.09	A
		2020	0.00 (0.00-0.00)	0	0.01	0	0.18	AB
		2021	0.00 (0.00-0.00)	0	0.00	0	0.06	B
	TBI							
		2018	0.02 (0.01-0.03)	0	0.07	0	1.00	AB
		2019	0.03 (0.02-0.03)	0	0.08	0	1.00	A
		2020	0.04 (0.01-0.06)	0	0.41	0	13.3	AB
		2021	0.02 (0.02-0.03)	0	0.08	0	0.78	B
	TI_{ADD}							
		2018	0.07 (0.05-0.09)	0	0.26	0	2.74	AB
		2019	0.09 (0.07-0.11)	0	0.29	0	5.48	A
		2020	0.11 (0.04-0.18)	0	1.13	0	35.80	AB
		2021	0.07 (0.06-0.08)	0	0.26	0	3.88	B
	TI_{UDD}							
		2018	0.06 (0.04-0.07)	0	0.20	0	2.74	AB
		2019	0.07 (0.06-0.09)	0	0.21	0	2.74	A
		2020	0.10 (0.03-0.16)	0	1.13	0	36.44	AB
		2021	0.06 (0.05-0.07)	0	0.21	0	2.13	B
